# ADDRESSING THE CHRONIC PAIN EPIDEMIC AMONG OLDER ADULTS IN UNDERSERVED COMMUNITY HEALTH CENTERS

**DOI:** 10.1093/geroni/igad104.1800

**Published:** 2023-12-21

**Authors:** Ana-Maria Vranceanu, Jonathan Greenberg, Katherine McDermott, Madison Ehmann, Nadine Lecvy, Julie Brewer, Christine Ritchie

**Affiliations:** Massachusetts General Hospital, Boston, Massachusetts, United States; Massachusetts General Hospital/Harvard Medical School, Boston, Massachusetts, United States; Massachusetts General Hospital/Harvard Medical School, Boston, Massachusetts, United States; Massachusetts General Hospital, Boston, Massachusetts, United States; Massachusetts General Hospital, Boston, Massachusetts, United States; Massachusetts General Hospital, Boston, Massachusetts, United States; Massachusetts General Hospital, Boston, Massachusetts, United States

## Abstract

Current nonpharmacological treatments are inadequate for addressing chronic pain among older adults from disadvantaged backgrounds. These treatments have been developed for affluent populations and are not implemented in underserved community health centers (CHCs) where most older adults with chronic pain seek care. We conducted 4 focus groups with 23 providers (e.g., primary care doctors, nurse practitioners, medical interpreters and support staff) and 4 focus groups with 15 English- and Spanish-speaking patients at a CHC. Our goals were to: 1) understand barriers and facilitators to sustained implementation of the GetActive+ mind-body activity program delivered by a nurse practitioner; 2) gather perceptions of program skills to facilitate cultural tailoring; and 3) gather information to support a future hybrid type 1 effectiveness implementation trial of GetActive+ versus usual care to improve pain outcomes. Qualitative analyses were conducted using a deductive-inductive approach and the Framework method. Questions were informed by Aaron’s implementation model and the Socioecological model. Results revealed multiple barriers at the individual (e.g., pain misconceptions, perceived bias, stigma, shame, treatment expectations and loneliness, language barriers, food and financial insecurity), health clinic (e.g., provider burnout, understaffed clinic, budget cuts, multiple languages) and environmental levels (e.g., lack of technology, lack of reliable transportation to clinic, billing challenges). Clinic providers felt unprepared to support older adults with chronic pain. Both patients and providers endorsed the need for, and skills of GetActive+. Nuanced differences between patient-provider and English-Spanish patients as well as modifications to the program manual, protocol, and interventionist training will be discussed.

